# Research on a Road Crack Detection Method Based on YOLO11-MBC

**DOI:** 10.3390/s25247435

**Published:** 2025-12-06

**Authors:** Jinhui Li, Xiaowei Jiang, Hui Peng

**Affiliations:** 1 College of Vehicle and Traffic Engineering, Henan University of Science and Technology, Luoyang 471003, China; jtljh@haust.edu.cn; 2 Henan Urban Transportation Planning and Design Research Institute Co., Ltd., Xuchang 461000, China; hnph202511@163.com

**Keywords:** road crack detection, YOLO11, multi-scale feature fusion, attention mechanism, loss function

## Abstract

To address the issues of low accuracy and high rates of false detection and missed detection in existing methods for pavement crack identification under complex road conditions, this paper proposes a novel approach named YOLO11-MBC, based on the YOLO11 model. A Multi-scale Feature Fusion Backbone Network (MFFBN) is designed to enhance the model’s capability to recognize and extract crack features in complex environments. Considering that pavement cracks often exhibit elongated topologies and are susceptible to interference from similar features like tree roots or lane markings, we combine the Bidirectional Feature Pyramid Network (BiFPN) with a Multimodal Cross-Attention (MCA) mechanism, constructing a novel BiMCNet to replace the Concat layer in the original network, thereby optimizing the detection of minute cracks. The CGeoCIoU loss function replaces the original CIoU, employing three distinct penalty terms to better reflect the alignment between predicted and ground-truth boxes. The effectiveness of the proposed method is validated through comparative and ablation experiments on the public RDD2022 dataset. Results demonstrate the following: (1) Compared to the baseline YOLO11, YOLO11-MBC achieves a 22.5% improvement in F1-score and an 8% increase in mAP50 by integrating the three proposed modules, significantly enhancing performance for complex pavement crack detection. (2) The improved algorithm demonstrates superior performance. Compared to YOLOv8, YOLOv10, and YOLO11, it achieves precision, recall, F1-score, mAP50, and mAP50-95 of 61%, 70%, 72%, 75%, and 66%, respectively, validating the correctness of our approach.

## 1. Introduction

By the end of 2024, China’s total highway mileage had reached 5,490,400 km, with expressways accounting for 190,700 km. This total mileage ranks first globally [1]. However, the pavement is the first component of the road structure to bear external forces, enduring loads from passing vehicles while also facing impacts such as temperature fluctuations, corrosion, and human damage. The intensification of these factors inevitably leads to road deterioration. In the “14th Five-Year Plan for Highway Maintenance Management Development,” the Ministry of Transport proposed leveraging digital technologies to advance highway maintenance and management techniques, accelerate the R&D of inspection equipment, and enhance the automation of road inspection and maintenance [2]. Pavement defect detection serves as a critical preliminary step in road maintenance, playing a pivotal role in ensuring safe and efficient daily transportation while fostering stable socioeconomic development [3].

Common pavement defects include longitudinal cracks, transverse cracks, and crocodile cracks. Early pavement crack detection methods primarily relied on traditional image processing techniques, encompassing both 2D and 3D approaches. Typical 2D methods, such as edge detection based on the Canny operator [4] and threshold segmentation using Otsu’s method [5], are highly sensitive to environmental factors like lighting variations and pavement conditions. These methods not only require frequent manual intervention to adapt to different scenarios—significantly reducing the level of automation and detection efficiency—but also tend to introduce biases during crack feature extraction, leading to frequent false positives and false negatives. Although 3D detection models process richer information, they are also susceptible to environmental interference, require complex denoising, and suffer from limited application scenarios. Moreover, these models are computationally intensive, inefficient, and demand high hardware specifications [6].

In recent years, with the continuous advancement of machine learning and artificial intelligence, computer vision has been increasingly applied in the field of object detection, which is primarily divided into two-stage and single-stage models. Two-stage models, typified by the R-CNN series [7], first generate candidate boxes and then perform secondary feature extraction and multi-task learning to achieve object detection. They have demonstrated outstanding performance in medical image lesion segmentation, 3D obstacle detection in autonomous driving, and military target detection in satellite imagery. Single-stage object detection models enable direct end-to-end prediction of both object category and location, offering faster detection speeds. Classic single-stage detection models include the SSD and YOLO series [8]. Among these, the YOLO series [9] stands as a quintessential single-stage model, achieving a remarkable balance between real-time performance and accuracy. It has become the mainstream tool for road crack detection.

Duo Ma et al. [10] proposed the YOLO-MF method based on an improved YOLOv3. They employed a PCGAN to generate realistic crack images and optimized the approach using accelerated algorithms and the Median Flow (MF) algorithm. However, although the PCGAN-generated data alleviates data scarcity, it cannot fully capture the diversity and complexity of real-world cracks, thereby limiting the model’s generalization capability. An Xue-Gang et al. [11] enhanced the detection accuracy for pavement defects by improving YOLOv4 with adaptive spatial feature fusion and modifications to the Focal Loss function. Sanchez et al. [12] annotated bounding boxes based on a nine-category list of damaged and undamaged objects. Beyond detecting cracks amidst background noise on asphalt surfaces, they designed six augmented scenarios by applying horizontal and vertical flipping to evaluate model performance. The YOLOv5 model demonstrated consistent detection of well-defined defects. Huantong Geng et al. [13] proposed the Selective Dynamic Feature Compensation YOLO (SDFC-YOLO) algorithm, which introduced a Dynamic Downsampling Module (DDM) to adaptively adjust the sampling positions of convolutional kernels during feature extraction, alongside a novel feature fusion method. However, integrating multiple sophisticated modules increased the complexity of the training process, demanding greater computational resources and time for parameter optimization. Zhang et al. [14] integrated the Convolutional Block Attention Module (CBAM) into YOLOv7 to enhance accuracy. However, relying exclusively on attention mechanisms can lead to false detections in complex road environments. Li Song et al. [15] proposed an improved lightweight road damage detection algorithm, YOLOv8-RD, which combines the strengths of CNN and Transformer architectures. By introducing the BOT module and a coordinate attention mechanism, the detection efficiency was improved. Although this model achieved better performance for small objects to some extent, its accuracy remained insufficient for detecting extremely fine cracks. Yuan, Hongshuai et al. [16] adopted the scale sequence feature fusion module and the triple feature encoder module from the ASF-YOLO architecture to enhance the detection of multi-scale cracks and improve target feature perception. They also incorporated the Coordinate Attention (CA) mechanism, embedding positional information into channel attention to bolster crack feature extraction. However, the increased model complexity resulted in slower detection speeds compared to other models. For lightweight applications, Xu Tiefeng et al. [17] proposed the DGE-YOLO-P crack detection model based on YOLOv8. They designed the C2f-DCNv3 module to enhance modeling capacity and reduce the dimensionality of input features, effectively decreasing the number of model parameters and computational complexity.

However, pavement cracks—characterized by weak textures, high aspect-ratio, and significant scale variations—are prone to being confounded with environmental noise such as oil stains, repair marks, and tree roots. As highlighted by Dong et al. [18] in their 2025 study on YOLO11-based bridge crack detection, YOLO series models still face “considerable difficulties” when detecting narrow, elongated cracks with low contrast, including significant background false positives and missed detections. These limitations indicate that practical applications of YOLO models remain constrained in this domain. As YOLO undergoes continuous updates and iterations, road crack detection must integrate crack-specific features while continually refining its network architecture. Currently, the YOLO algorithm undergoes continuous optimization and version updates. In 2024, the Ultralytics team introduced the YOLO11 [19] version, which achieves high detection accuracy and a relatively lightweight network architecture while maintaining real-time performance, making it suitable for targeted improvements in detecting road crack objects. Zhang, Y. et al. [20] proposed the GLNET-YOLO framework based on cross-modal deep feature fusion, integrating visible and infrared image features. This framework extends the YOLO11 architecture by introducing the FM module for global feature fusion and enhancement, and the DMR module for local feature separation and interaction. It significantly improves detection accuracy and algorithm robustness under low-light and complex background conditions, with its effectiveness further validated on the KAIST dataset.

Based on this, this paper analyzes the YOLO11 model and optimizes it through three key improvements. A Feature Fusion Backbone Network (MFFBN) is designed to enhance the recognition and extraction of pavement crack features in complex environments. The BiFPN weighted bidirectional feature pyramid network is combined with the MCA multimodal cross-attention mechanism to propose the BiMCNet (Multi-Channel Attention Bifurcate Network) to replace the Concat layer in the original network architecture, thereby optimizing the model’s detection capability for fine cracks. The CGeoCIoU (Crack Geometrically Improved Complete IoU) replaces the original model’s CIoU, and by adjusting model accuracy through three distinct penalty terms, a novel pavement crack detection method—YOLO11-MBC (YOLO11-MFFBN-BiMCNet-CGeoCIoU)—is proposed. This addresses the issues of low recognition accuracy and high false positive/miss rates in complex road conditions.

## 2. Materials and Methods

### 2.1. Basic YOLO11 Model Architecture

**Figure 1:**
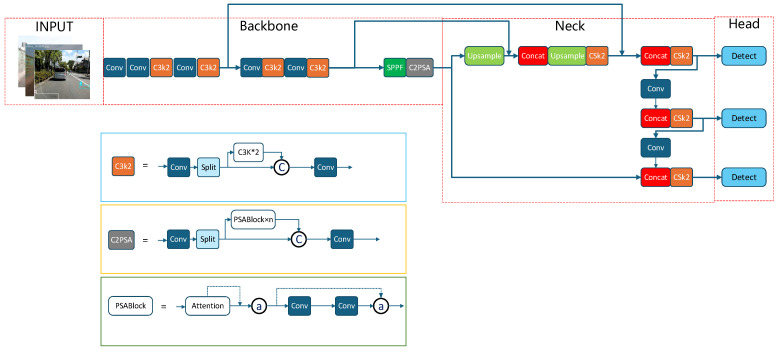
Yolo11 original model.

YOLO11, released by Ultralytics on 30 September 2024, is the latest version in the YOLO series of real-time object detection algorithms. It achieves significant improvements in speed, accuracy, and efficiency compared to its predecessors. Developed through a series of optimizations based on YOLOv8 [21], the model incorporates several innovative enhancements, including the C3k2 module, the Spatial Pyramid Fast Pooling (SPFF) module, and the Cross-Stage Partial Spatial Attention (C2PSA) mechanism. The C3k2 module employs a dual-branch design: a 3 × 3 convolutional branch captures local features, while a 1 × 1 convolutional branch facilitates channel interaction for feature extraction. The SPFF module fuses spatial features at different granularities through multi-scale pooling and aggregates multi-scale contextual information via multiple max-pooling operations (e.g., three repetitions of 5 × 5 pooling), thereby reducing computational complexity compared to traditional Spatial Pyramid Pooling (SPP) modules. The C2PSA mechanism extracts features in parallel using multi-scale convolutional kernels, generating multi-scale feature maps that enhance the model’s focus on critical regions. The basic YOLO11 model architecture is illustrated in Figure 1.

However, the basic YOLO11 model faces several challenges in road crack detection: (1) Although the C3k2 module excels in feature extraction, its excessive focus on local features may cause it to overlook the overall continuity of cracks when processing such fine and complex textures, leading to inaccurate detection results. (2) The width and length of cracks can vary significantly, and the SPFF module may struggle to adapt to these variations, resulting in incomplete crack detection. (3) While the C2PSA mechanism enhances the perception of target details, it may be inadequate for handling the substantial variations in crack shape and position, leading to detection inaccuracies. Additionally, the high computational complexity of the C2PSA mechanism may compromise the model’s real-time performance.

### 2.2. YOLO11—Improved Multi-Bid Frameworks

**Figure 2:**
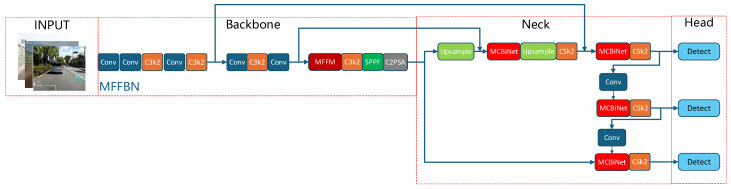
YOLO11-MBC model.

To address the shortcomings of C3K2 in poor feature extraction for minute pavement cracks, SPPF module’s incomplete crack detection coverage, and C2PSA’s high computational complexity, improvements were made to propose the YOLO-MBC integrated network architecture, as shown in Figure 2. The main enhancements include:

1. Design of the MFFBN Backbone: Since cracks typically occupy less than 5% of the image area and are easily obscured by repeated downsampling, we designed a Feature Fusion Backbone Network (MFFBN). This backbone integrates the MFFM [22] with the principles of the ECA mechanism [23] to enhance the perception of target boundaries and minute defects. It enables the model to focus on critical regions within the target area while filtering out noise such as road surface reflections and oil stains in the spectral dimension. This improvement ensures accurate identification of road cracks and potholes in complex detection environments.
2. Design of the BiMCNet Module: Given that cracks on road surfaces often exhibit slender, elongated topologies and are susceptible to occlusion and disruption from uneven lighting, we designed the BiMCNet architecture. This structure is centered on the BiFPN module and incorporates the Multimodal Cross-Attention (MCA) mechanism. MCA allows queries from local breakpoints to align with globally semantic keys, locating pixels with consistent orientation to achieve spatial stitching. Consequently, it reconstructs occluded, elongated cracks in the spatial dimension while suppressing the influence of false targets such as lane markings, repair marks, and tree roots on the detection results.
3. Introduction of CGeoCIoU function: When the crack aspect ratio exceeds 10:1 and orientation is arbitrary, CIoU still treats the target as an “axis-aligned bounding box.” The CGeoCIoU loss function is introduced to enhance the model’s perception of crack boundaries.

### 2.3. Design of the MFFBN Backbone

Individual cracks typically occupy less than 5% of an image’s area. These defects are characterized by minute dimensions and low contrast, making them easily overlooked during deep convolution processes. Additionally, complex road surfaces may exhibit crack-like textures, which blur the boundaries of target cracks and compromise the model’s detection accuracy and robustness. Although YOLO11 enhances feature expression through multiple residual connections and local convolutional operations, it lacks sufficient sensitivity to small objects and specialized scale adaptation mechanisms. Furthermore, real-world roads exhibit imbalanced distributions of defect categories due to usage patterns and environmental factors.

**Figure 3:**
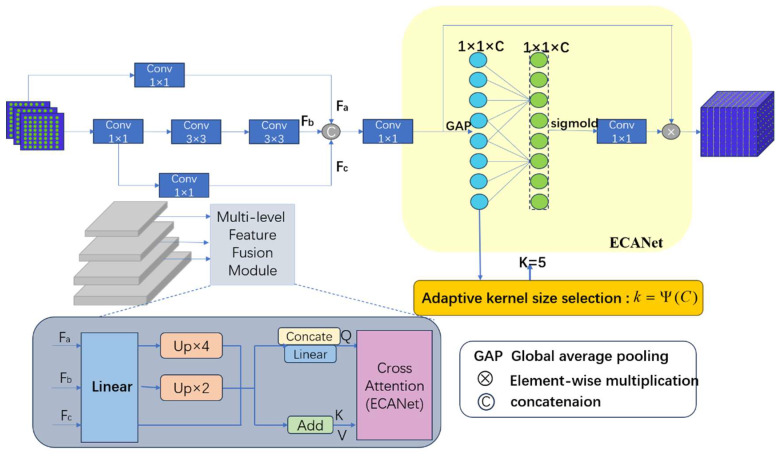
Proposed multi-scale feature fusion module MFFM.

Therefore, this paper proposes a Feature Fusion Backbone Network (MFFBN), which integrates the C3k2 module with a custom-designed Feature Fusion Module (MFFM) to achieve refined feature extraction. This architecture focuses on detecting minute cracks while simultaneously mitigating class imbalance, thereby enabling effective detection of road surface crack defects. The structure of the MFFM is illustrated in Figure 3, where the left part performs multi-scale cross-layer fusion and the right part conducts dynamic channel weight adjustment.

(1) Multi-scale cross-layer fusion primarily achieves this by simultaneously preserving high-resolution edge localization information and deep semantic discrimination information, specifically accomplished using multiple 1 × 1 and 3 × 3 convolutions where 1 × 1 convolutions capture fine local features—such as minor road cracks—while eliminating redundant information. Conversely, 3 × 3 convolutions expand the receptive field to extract contextual features. Through successive convolutions, these operations ultimately merge shallow-level semantic information with high-frequency features across varying scales, synthesizing a unified representation. The feature fusion process is described by Equation (1):

(1) $$Z=C_{1\times 1}\left(Concat\left(F_{a},F_{b},F_{C}\right)\right)$$

In the above equation, $F_{a}\in R^{\frac{H}{32}\times \frac{W}{32}\times 1024}$ represents features derived from the upper layer output after one convolution operation, $F_{b}\in R^{\frac{H}{32}\times \frac{W}{32}\times 1024}$ providing additional local detail information; $F_{C}\in R^{\frac{H}{32}\times \frac{W}{32}\times 1024}$ denotes features processed through one convolution and two convolutions to deliver higher-level semantic information; represents features obtained after two convolutions, supplying surrounding semantic information. Finally, $C_{1\times 1}\left(•\right)$ performs batch normalization and the convolution operation of the activation layer. The three feature maps are concatenated along the channel dimension. $Z\in R^{\frac{H}{32}\times \frac{W}{32}\times 1024}$ is the output obtained after applying a convolutional operation to the concatenated feature. Through multi-scale cross-layer fusion of features, the model can better capture the boundary and detailed characteristics of small objects such as minor cracks on complex roads. This significantly enhances the model’s ability to express features across different scales, making it more suitable for feature extraction tasks that might be overlooked in complex scenes.

(2) Dynamic channel weight adjustment: In the task of road crack detection, the proposed Feature Fusion Module (MFFM) introduces an innovative dynamic channel weight adjustment mechanism inspired by the Effective Channel Attention (ECA) mechanism. This mechanism replaces cumbersome fully connected layers with efficient convolutional operations, thereby reducing model complexity while maintaining computational efficiency. This design optimizes the feature extraction process, enhancing the model’s ability to recognize pavement crack features.

A key advantage lies in its adaptive enhancement of crack-related feature channels while suppressing interference from non-target features such as background noise and lighting variations. This is crucial for enhancing the model’s sensitivity to subtle cracks, particularly when crack features resemble or are indistinguishable from the surrounding environment. Furthermore, the mechanism excels at addressing class imbalance by automatically boosting feature weights for underrepresented classes. This prevents these important but scarce categories from being overwhelmed by abundant information from common classes during feature learning.

### 2.4. Design of the BiMCNet Module

**Figure 4:**
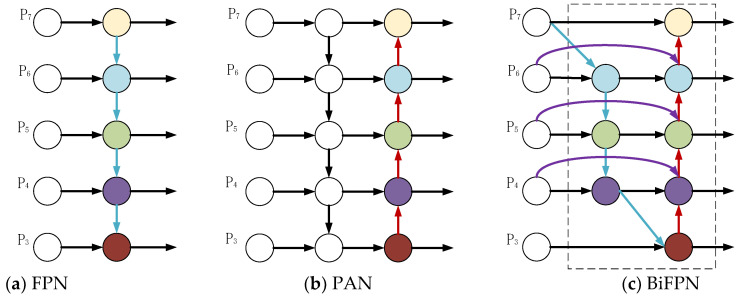
FPN, PAN, and BiFPN network structures.

To enhance the performance of pavement crack detection, particularly for identifying fine cracks, the Bidirectional Feature Pyramid Network (BiFPN) module was introduced and applied to the YOLO11-MBC pavement crack detection model. The Neck network serves as a critical component within object detection frameworks, efficiently reorganizing multi-level feature maps output by the backbone network. This process fuses high-resolution spatial details with low-resolution semantic abstractions, thereby providing the detection head with multi-scale representations that enhance discriminative power. Traditional Feature Pyramid Networks (FPN) propagate semantic information solely through unidirectional top-down paths. PANet introduces bottom-up branches to supplement spatial details yet still suffers from information loss and insufficient balance. BiFPN (Bidirectional Feature Pyramid Network), proposed in EfficientDet [21,24], achieves more comprehensive and flexible multi-scale information exchange within the same computational budget through bidirectional cross-scale connections and a learnable weighting fusion mechanism. Embedding BiFPN into the YOLO11 neck significantly enhances the model’s detection accuracy and robustness for cross-scale pavement defects—ranging from minute cracks to large-area damage—without substantially increasing inference latency. (See Figure 4).

Although the PAN-FPN architecture in YOLO11 possesses bidirectional multi-scale fusion capabilities, the repeated upsampling and downsampling processes generate feature redundancy and additional computational overhead, leading to increased computational demands. BiFPN partially alleviates this computational pressure through learnable weight fusion; however, its receptive field remains confined to local neighborhoods. This limitation hinders the capture of global context, resulting in inadequate suppression of road surface background noise. Furthermore, BiFPN lacks a fine-grained filtering mechanism, exhibiting weak selectivity for the spatial orientation features of sub-pixel cracks and small potholes. Consequently, it suffers from representation gaps and localization errors in complex textured backgrounds.

**Figure 5:**
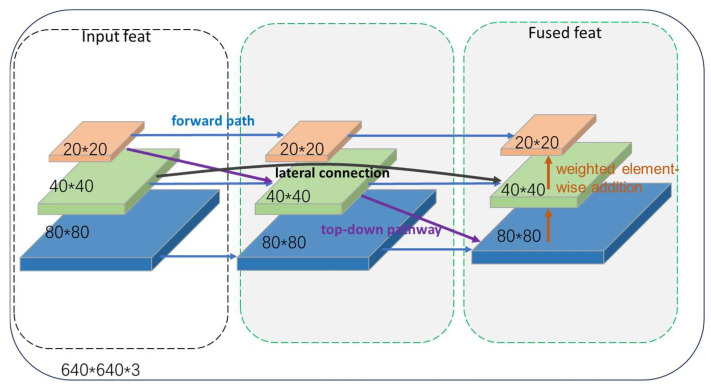
BiMCNet principle.

To dynamically optimize multi-scale feature processing, enhance target discrimination and contextual awareness, strengthen the capture of spatial orientation features for small cracks and potholes, and maintain lightweight and efficient computation, a novel structure named BiMCNet is embedded into the YOLO11 model. Its specific implementation steps are shown in Figure 5. When a feature map has only one input path and undergoes no further processing, it typically contributes little to the feature network. For feature maps with two input paths, if they are at the same scale, an additional path is added from the backbone features and fused with the features from the PAN path. This processing method achieves enhanced fusion without introducing extra parameter costs.

In traditional Feature Pyramid Networks (FPN), input features are typically treated equally without considering their varying contributions to the output. In contrast, BiMCNet optimizes the contribution of features at different resolutions by assigning unique weights to each channel, thereby improving the feature fusion effect. Its definition is given by Equation (2).

(2) $$c=\sum_{i}\frac{w_{i}}{\sum_{j}w_{j}+\varepsilon }I_{i}$$

The variable c represents the output, $I_{i}$ denotes the inputs at each node, and $w_{i}$ signifies the cumulative weights of these inputs, which are learnable parameters. To ensure computational stability, a small value e=0.0001 is introduced. Additionally, each bidirectional path (including both top-down and bottom-up pathways) is treated as an independent module and can be reused to enhance feature integration. Taking the *i*-th level as an example, the two feature fusion formulas in BiMCNet are shown in Equation (3) and Equation (4), respectively.

(3) $$P_{i}^{td}=Conv(\frac{w_{1}p_{i}^{in}+w_{2}p_{i+1}^{in}}{w_{1}+w_{2}+\varepsilon })$$

(4) $$p_{i}^{out}=Conv(\frac{w_{1}p_{i}^{in}+w_{2}p_{i}^{td}+w_{3}p_{i-1}^{out}}{w_{1}+w_{2}+w_{3}+\varepsilon })$$

**Figure 6:**
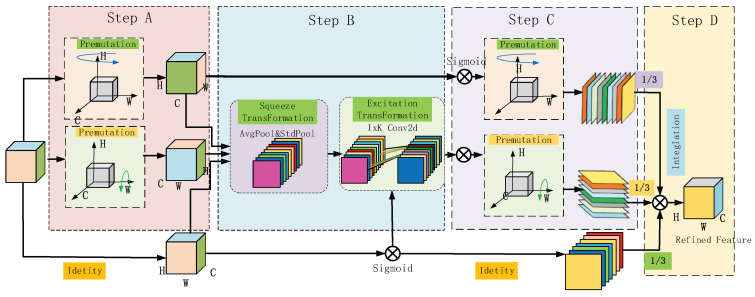
MCA structure diagram.

To further enhance contextual information capture and feature selectivity, the BiMCNet architecture also integrates the MCA multimodal cross-attention mechanism. As shown in Figure 6, multimodal cross-attention (MCA) serves as a core mechanism for fusing multi-source heterogeneous data. Its objective is to automatically establish cross-modal correlations within a unified semantic space, achieving information filtering and fusion through learnable attention weights. Taking visual-language tasks as an example, MCA establishes bidirectional dependencies between image and text features: on one hand, textual queries guide the model to focus on semantically relevant regions within images; on the other hand, image context reciprocally enhances the interpretation of textual semantics. In implementation, features from each modality are first mapped into a unified Query-Key-Value (Q-K-V) representation. For image-text pairs, visual features serve as the Query, while linguistic features act as both Key and Value. Attention weights are computed by measuring the similarity between Query and Key (typically using dot product or cosine similarity), followed by softmax normalization. These weights directly determine the weighted aggregation of Values, dynamically amplifying key information.

In the figure, C×H×W the feature tensor *F* outputs a processed feature tensor *F*′ of the same size as *F*. The output process involves two steps: coordinated attention embedding and coordinated attention generation. Below, I will describe the output process. The fusion of the MCA multimodal cross-attention mechanism can be divided into four steps:

Step A: First, perform global pooling on the input vector *F* to obtain global feature information. The input image is encoded along both horizontal and vertical spatial directions before undergoing global pooling. This yields one-dimensional vector features in the height and width directions, enabling subsequent global feature extraction while preserving coordinate information. For the convolutional operation *F*_1_, the encoding of the cth channel at the height and weight positions can be described by the following equation:

(5) $$z_{c}^{h}(h)=F_{1}(f_{c})=\frac{1}{W}\underset{0<n<W}{\sum }f_{c}(h,n)$$

(6) $$z_{c}^{W}(W)=F_{1}(f_{c})=\frac{1}{H}\underset{0<m<H}{\sum }f_{c}(m,w)$$

Step B: This transformation establishes long-range dependencies along the horizontal direction, enabling attention to focus on lateral global features while preserving precise positional coordinates vertically. Subsequently, the two feature maps from the global receptive field are concatenated and fed into a 1 × 1 convolution. Let denote the 1 × 1 convolution operator and R denote the nonlinear activation function, as detailed in the following equation:

(7) $$Z=R(F_{2}(\left[z^{h},z^{w}\right]))$$

Step C: The concatenated feature tensor [$Z^{h},Z^{w}$] obtained in the previous step is an intermediate feature map containing horizontal and vertical feature information. Two 1 × 1 convolution operators are used to split Z into two feature tensors and in the horizontal and vertical directions, respectively. These are transformed into two tensors matching the size of the input feature in both horizontal and vertical dimensions. Subsequently, they are fed into the sigmoid activation function, defined by the following equation:

(8) $$y^{h}=\sigma (F_{h}(Z^{h}))$$

(9) $$y^{w}=\sigma (F_{w}(Z^{w}))$$

Step D: Finally, we concatenate these feature tensors along two spatial dimensions to obtain the attention weights for the channel features, treating them as the output feature map F′. The entire computation process is summarized in the following equation:

(10) $$F_{c}^{\prime }(n,m)=f_{c}(n,m)y_{c}^{h}(n)y_{c}^{w}(m)$$

The attention module not only acquires channel information during encoding but also incorporates positional information. Specifically, the coordinate information retained in the vertical direction enables the network to precisely determine the starting and ending positions of cracks or potholes along the longitudinal axis. Meanwhile, the horizontal dimension facilitates the detection of elongated cracks extending laterally or contiguous damaged areas. This significantly enhances the localization accuracy of multi-scale cracks.

### 2.5. Introduction of the CGeoCIoU Loss Function

Since typical road surface cracks exhibit aspect ratios exceeding 10:1 and arbitrary orientations, the original model’s CIoU still treats targets as “axis-aligned rectangles.” Therefore, the CGeoCIoU (Crack Geometrically Improved Complete IoU) loss function is introduced to enhance the model’s perception of crack boundaries.

YOLO11 employs CIoU as its bounding box loss function, which accounts for factors such as the aspect ratio and center point distance between target boxes. Its calculation is defined by Equations (11)–(13), where represents the Euclidean distance between the centers of two boxes, c denotes the minimum diagonal length of the bounding box, and is a weight parameter used to measure the consistency of width and height between the two boxes.

(11) $$CIoU=IoU-(\frac{\rho^{2}(box_{1},box_{2})}{c^{2}}+av)$$

(12) $$a=\frac{v}{(1-IoU)+v}$$

(13) $$v=\frac{4}{\pi^{2}}{\left(\arctan\frac{\varpi_{box1}}{h_{box1}}-\arctan\frac{\varpi_{box2}}{h_{box2}}\right)}^{2}$$

In crack detection, due to the characteristics of cracks themselves—such as being slender, highly directional, and having irregular boundaries—traditional IoU-based bounding box loss functions often fail when aligning complexly shaped or inconsistent-scale crack targets. While CIoU, adopted in YOLO11, considers factors like center point distance, aspect ratio, and bounding box distance, it still struggles to address the following issues:

- Although the centers of the two boxes coincide, there are noticeable differences in corner positions, orientations, or boundary contours;
- Angular deviations in elongated crack targets significantly impact detection quality, yet CIoU fails to capture this “rotational misalignment”;
- Smaller crack targets are sensitive to geometric variations, yet uniform weighting may impose unfair penalties.

**Figure 7:**
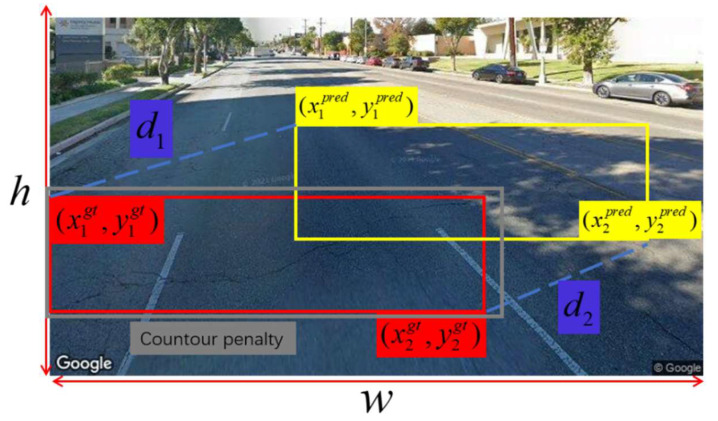
CGeoCIoU regression loss.

To address this, an improved direction-aware geometric IoU loss function is proposed based on CIoU. The enhanced diagram is shown in Figure 7, incorporating three optimization terms as follows:

1. Corner Distance Penalty:

Measures the Euclidean distance difference between the top-left and bottom-right corners of the predicted box and the ground truth box, respectively, and incorporates it as a penalty term:

(14) $$d_{1}=\sqrt{{\left(x_{lt}^{pred}-x_{lt}^{gt}\right)}^{2}+{\left(y_{lt}^{pred}-y_{lt}^{gt}\right)}^{2}}$$

(15) $$d_{2}=\sqrt{{\left(x_{rb}^{pred}-x_{rb}^{gt}\right)}^{2}+{\left(y_{rb}^{pred}-y_{rb}^{gt}\right)}^{2}}$$

2. Contour Alignment Penalty:

Based on the minimum bounding contours of two frames, a boundary consistency metric is introduced:

(16) $$ContourPenalty=1-\frac{Area\left(B_{pred}\cap B_{gt}\right)}{Area\left(Hull\left(B_{pred}\cup B_{gt}\right)\right)}$$

Enalizes cases where the predicted box edge shape significantly deviates from the actual box, particularly effective for slender targets such as cracks.

3. Dynamic Weighting Strategy:

To prevent small targets from being overlooked, introduce target-aware weight adjustment:

(17) $$\omega_{i}=\frac{\lambda }{1+IoU}$$

Here, λ is the regularization coefficient, which increases the weight of the error term as the IoU decreases. In summary, the final loss function is defined as:

(18) $$CGeoCIoU=CIoU+\omega_{1}\frac{d_{1}^{2}}{\omega^{2}+h^{2}}+\omega_{2}\frac{d_{2}^{2}}{\omega^{2}+h^{2}}+\omega_{3}\cdot ContourPenalty$$

This loss function significantly enhances the representation capabilities for directionality, detailed position alignment, and boundary contour morphology in crack detection while preserving the original CIoU stability. Its effectiveness will be further validated in Section 4.1 below.

## 3. Experiment

### 3.1. Dataset

**Figure 8:**
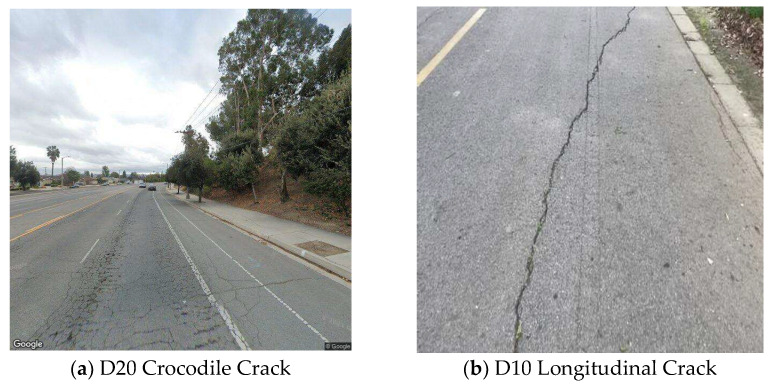

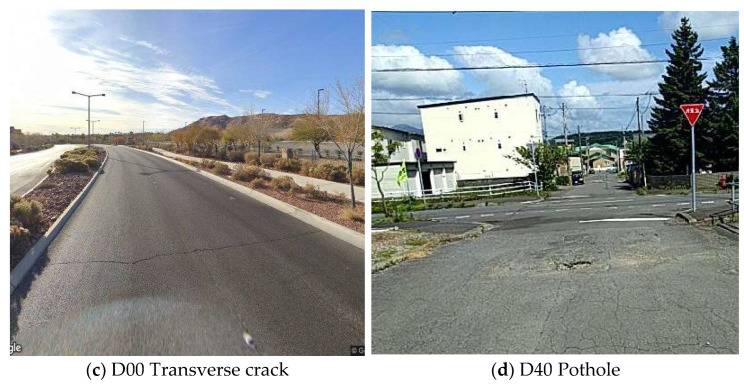
Crack types in the experimental dataset.

For the road crack detection task, this paper employs the open-source RDD2022 dataset for model training and validation. Released by the University of Tokyo, RDD2022 comprises 47,420 road surface images collected from Japan, India, the Czech Republic, Norway, the United States, and China, with over 55,000 annotated instances of road damage. Due to severe class imbalance in the dataset, only four categories were retained: D00 (transverse cracks), D10 (longitudinal cracks), D20 (crocodile cracks), and D40 (potholes). Illustrations of these defect categories are shown in Figure 8. During data preprocessing, images with monotonous content, indistinguishable defects, or erroneous annotations were filtered out.

Additionally, annotations with a bounding box area ratio of less than 0.05% were automatically removed from the dataset. From the remaining samples, 41,000 images were randomly selected to form the final dataset. The distribution of the four defect categories among these images is summarized in Table 1. The dataset was then split into training, validation, and test sets in an 8:1:1 ratio (with random seed 42), resulting in 32,800 images for training, 4100 for validation, and 4100 for testing.

### 3.2. Experimental Setup

The hardware configuration featured a 64-bit Windows 11 operating system, an Intel^®^ Core™ i7-12700H central processing unit, and an NVIDIA GeForce RTX 3060 graphics processing unit. The system was equipped with 64 GB of RAM and 12 GB of GPU memory. Model training was conducted using the PyTorch 1.13.1 deep learning framework (see Table 2).

This study configured the experimental parameters by adjusting training parameters according to the practical road crack detection task and the experimental dataset. During the training phase, Stochastic Gradient Descent (SGD) was adopted for parameter optimization, with an initial learning rate of 0.01 and cosine annealing to 1 × 10^−4^, a momentum of 0.937, 150 epochs (early stopping patience = 30), a batch size of 32, and weight decay of 5 × 10^−4^, as listed in Table 3.

### 3.3. Evaluation Indicators

Common metrics for evaluating model performance include precision, recall, and mean average precision (mAP). The specific definitions of each metric are as follows:

1. Precision

Precision is defined as the ratio of the number of samples predicted as positive by the model to the number of samples that are actually positive. The formula for precision (19) is as follows:

(19) $$\mathrm{Precision}=\frac{\mathrm{TP}}{\mathrm{TP}+\mathrm{FP}}$$

Among these, TP denotes True Positive, representing samples accurately predicted by the model, while FP denotes False Positive, representing counterexamples incorrectly predicted as positive by the model.

2. Recall Rate

Recall rate is defined as the ratio of all actual positive samples to the number of positive samples accurately identified by the model. The formula for calculating recall rate (20) is as follows:

(20) $$\mathrm{Recall}=\;\frac{\mathrm{TP}}{\mathrm{TP}+\mathrm{FN}}$$

Among these, FN denotes false negatives, representing samples that are actually of the positive class but are classified by the model as belonging to the negative class.

3. F1 Score

The F1 score is the harmonic mean of precision and recall, used to measure the balance between precision and recall in a model. Its calculation formula (21) is as follows:

(21) $$F_{1\_\mathrm{score}}=\frac{2\times \mathrm{Precision}\times \mathrm{Recall}}{\mathrm{Precision}+\mathrm{Recall}}$$

4. Average Precision and Mean Average Precision

Average Precision is a metric for evaluating the detection performance of individual categories. A higher value indicates better classifier performance. It is calculated based on the Precision-Recall Curve (PR curve), which illustrates how a model’s precision and recall change as the threshold varies. Average Precision represents the area under this curve. The formula is as follows:

(22) $$\mathrm{AP}=\int_{0}^{1}\mathrm{Precision}(\mathrm{Recall})d\mathrm{Recall}$$

The mean average precision is defined as the average of all mean average precisions. Typically, it is computed across different Intersection over Union (IoU) thresholds, as shown in Equation (23).

(23) $$\mathrm{mAP}=\frac{1}{N}\int_{0}^{1}\mathrm{Precision}(\mathrm{Recall})d\mathrm{Recall}$$

Among these, N represents the total number of categories in the dataset; mAP denotes the average precision of all predicted objects across categories; mAP50 indicates the mAP value at an IoU threshold of 0.5; mAP50-95 reflects the mAP values at IoU thresholds ranging from 50 to 95.

5. Frame Rate

Frame rate (Frames Per Second, FPS) indicates the number of frames a model can process per second, serving as a key metric for evaluating model performance. A higher FPS value signifies faster model inference speed. The calculation Formula (24) is as follows:

(24) $$\mathrm{FPS}=\frac{1}{\mathrm{Inference}\;\mathrm{Time}\;\mathrm{Per}\;\mathrm{Image}}$$

6. Giga Floating-point Operations Per Second (GFLOPs)

Giga Floating-point Operations Per Second (GFLOPs) is a metric for measuring a model’s computational intensity, representing the number of floating-point operations executed per second.

## 4. Result and Discussion

### 4.1. Comparative Analysis of CGeoCIoU Function Experiments

To analyze the impact of the three penalty terms in the CGeoCIoU loss function on detection results, we investigated the effects of different weight coefficients $\omega_{1}$, $\omega_{2}$ and $\omega_{3}$, and their influence on the model’s detection performance. By assigning reasonable weight coefficients to the three distinct penalty terms, we conducted comparative simulation experiments under varying weight coefficients to evaluate the matching between predicted boxes and ground truth boxes. The simulation results are presented in Table 4.

As shown in the table, the improved CGeoCIoU loss function demonstrates overall improvements over the baseline CIoU. At $\omega_{1}$ = 0.6 and $\omega_{2}$ = 0.4, its accuracy reaches approximately 54%, representing a 4.9% increase over the baseline model. Building upon this, when $\omega_{3}$ = 0.7, average precision reaches approximately 55%, achieving a 6.4% improvement over the baseline model. Therefore, the final parameters $\omega_{1}$ = 0.6, $\omega_{2}$ = 0.4, and $\omega_{3}$ = 0.7 were selected, achieving a better balance between the shape and positional differences between predicted and ground-truth bounding boxes.

### 4.2. Analysis of Ablation Experiment Results

Ablation studies were conducted to delineate the specific contributions of each proposed improvement to the overall model performance. In the context of pavement defect detection, these experiments allow for an in-depth analysis of the roles played by the MFFM and BiMCNet modules. By employing a controlled variable approach to examine the impact of individual components, this methodology prevents the blind stacking of enhancement modules, which could otherwise increase computational complexity or induce overfitting. This systematic comparison provides valuable insights for subsequent model refinement. In this paper, we trained and tested on 41,000 images using the original YOLO11 model alongside various improvement strategies and module combinations. The results are summarized in Table 5. It should be noted that all frame rate metrics were obtained on a Tesla A100 cloud node and are intended solely for relative comparison among the different methods.

(1) Comparison of Different Improvement Strategies

To systematically validate the specific contributions of the three proposed modules—MFFm, BiMCNet, and CGeoCIoU—to pavement crack detection performance, ablation studies were sequentially conducted based on the YOLO11 baseline. Analysis of the tabulated results leads to the following conclusions:

When individually incorporated, each of the three modules proves effective for complex pavement crack detection. With only a slight increase in computational load and a minor decrease in inference speed, the MFFM, BiMCNet, and CGeoCIoU modules improve the F1-score by 8%, 5%, and 9%, respectively, and increase the mean average precision (mAP50) by 3%, 4%, and 2%, compared to the baseline model. All pairwise combinations of these modules also lead to accuracy improvements, confirming the effectiveness of the proposed strategy. Ultimately, the complete YOLO11-MBC model achieves a 22.5% improvement in F1-score and an 8% increase in mAP50 over the baseline. (Modules adopted are marked with “√” in Table 5). The ablation studies verify that the proposed method effectively enhances the model’s capability for detecting complex pavement cracks.

(2) Comparison Before and After Improvement

**Figure 9:**
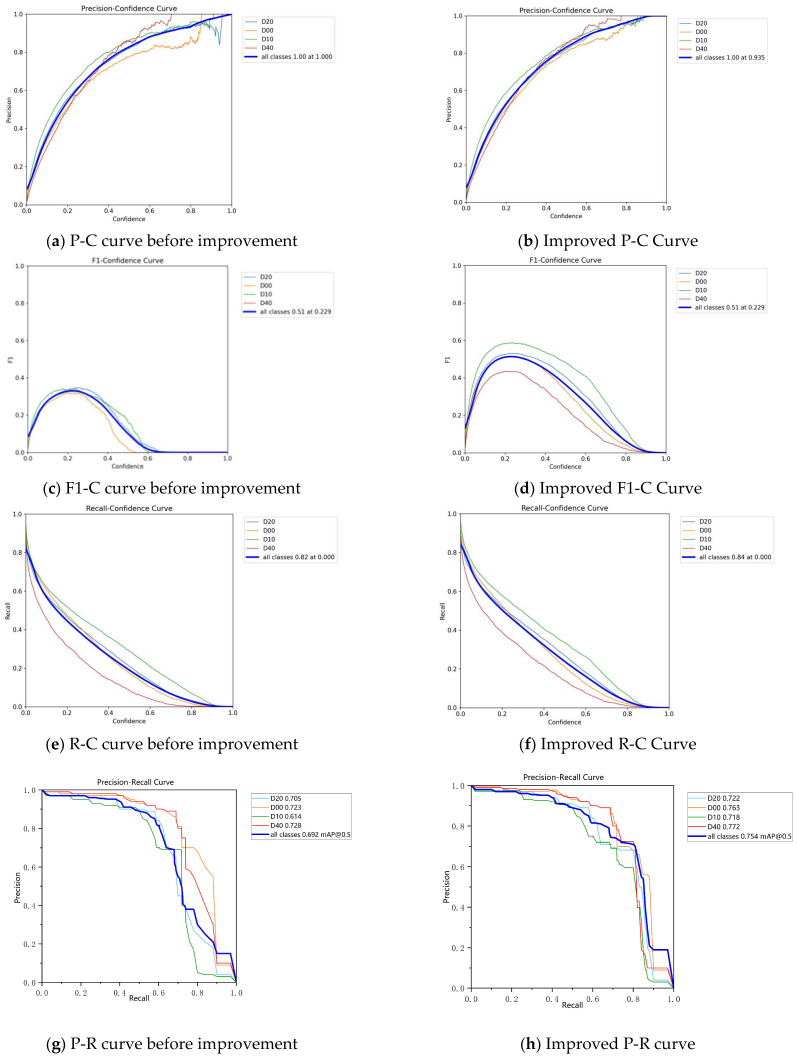
Comparison of indicators of various pavement crack categories.

Figure 9 shows the simulation results for various indicators under different pavement crack conditions. These include four categories: D00 (transverse crack), D10 (longitudinal cracks), D20 (crocodile cracks), and D40 (pothole). The “all classes” metric provides an overall assessment of the model’s performance in detecting these four distinct crack types, evaluated through precision-confidence curves (P-C curves), F1-confidence curves (F1-C curves), Recall-Confidence Curve (R-C curve), and Precision-Recall Curve (P-R curve).

Comparing Figure 9a,b reveals the optimized P-C curve. The overall upward shift indicates that the optimized model achieves higher precision at various confidence thresholds, demonstrating enhanced precision capabilities. Similarly, the optimized curves in Figure 9c,f,h (corresponding to Figure 9d,f,h) all exhibit an overall upward shift, signifying an overall improvement in model performance. In summary, the improved model outperforms the baseline model across all metrics.

**Figure 10:**
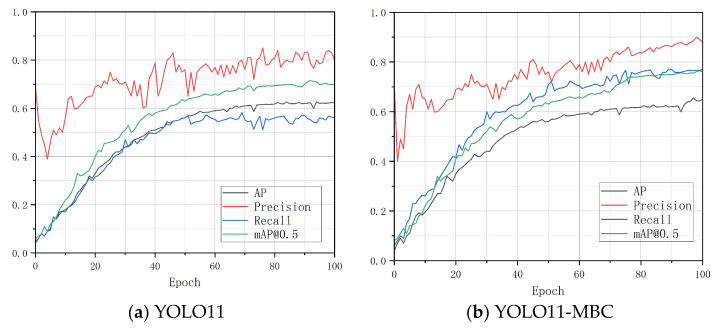
Accuracy curve on the RDD2022 dataset.

Figure 10 provides a visual comparison between the YOLO11-MBC model and the baseline YOLO11 model on several key accuracy metrics. Figure 10a shows the performance evolution of YOLO11 during training, and Figure 10b shows that of YOLO11-MBC. The horizontal axis indicates the number of training epochs, and the vertical axis represents the detection accuracy metrics. The curves in different colors correspond to recall, precision, average precision (AP), and mAP50, respectively.

As shown in the figure, YOLO11-MBC surpasses the baseline YOLO11 model across all evaluation metrics. As the number of training epochs increases, YOLO11-MBC demonstrates a more stable and pronounced upward trend in recall, precision, AP, and mAP50. These results indicate that the proposed improvements effectively enhance the detection capability of the model and verify the superior performance of YOLO11-MBC in the road crack detection task.

**Figure 11:**
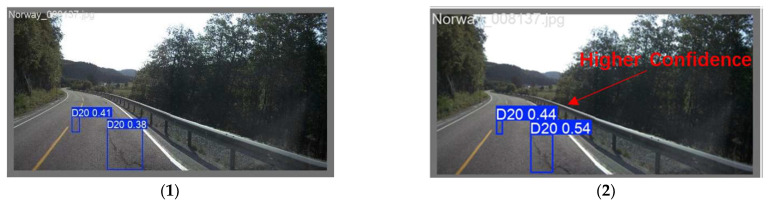

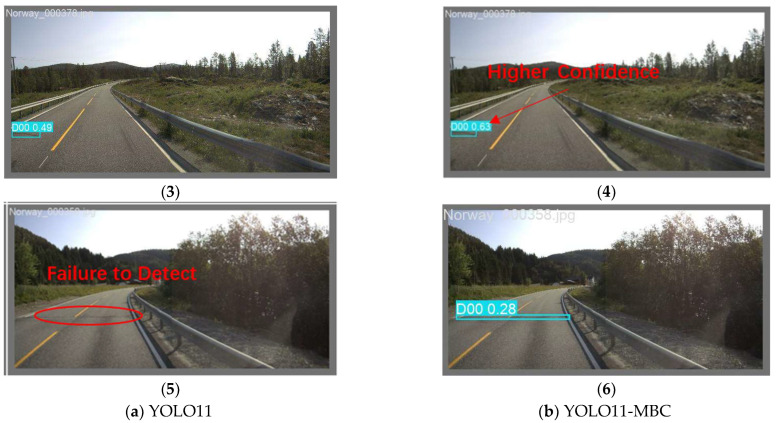
Visualization of detection results. (**1**–**6**) denote typical detection cases; (**a**) YOLO11 results, (**b**) corresponding YOLO11-MBC results.

Figure 11 presents a visual comparison of the performance between the original and the improved model on the RDD2022 dataset. It is evident that the improved model achieves significant performance enhancement. Specifically, in Figure 11(a1,b2), the original model exhibits insufficient sensitivity to small D20-type defects, leading to missed instances. This reflects its limitations in feature extraction. In contrast, the improved model successfully identifies these previously missed cracks, demonstrating its enhanced capability for localizing small objects. In Figure 11(a5,b6), when confronted with complex scenarios such as slender crack topologies and uneven lighting, the original model shows lower recognition accuracy, further validating the superior capability of the improved model in handling occlusions. Moreover, in Figure 11(a3,b4), the improved model demonstrates more precise localization of potholes, indicating its enhanced identification accuracy. In summary, the improved model achieves significant gains, enabling more accurate identification of road defects, precise bounding, and reliable classification, thereby enhancing its practicality in real-world road applications.

### 4.3. Comparative Analysis of Different Algorithms

**Figure 12:**
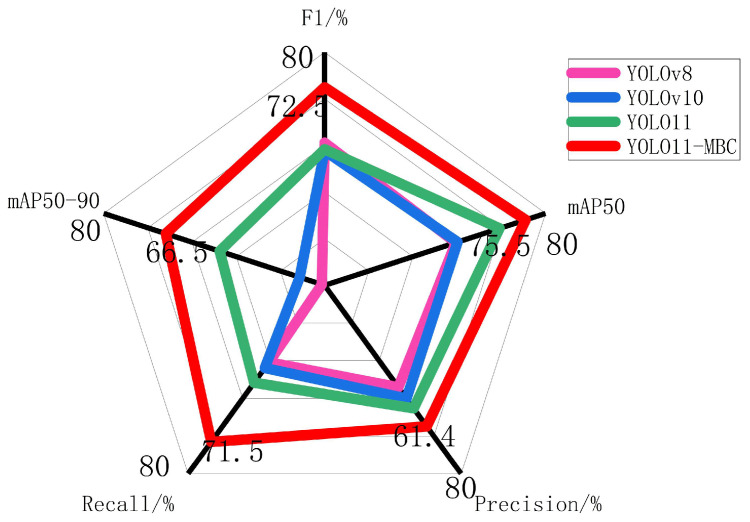
Comparative experiment of different algorithms.

To validate the detection performance of the proposed method, comparative experiments were conducted between the improved YOLO11-MBC model and mainstream object detection algorithms, including YOLOv8, YOLOv10, and the baseline YOLO11. Table 6 presents the detection results of these algorithms, while Figure 12 provides a radar chart visualization of the performance comparison. The results demonstrate that YOLO11-MBC exhibits outstanding detection performance. It achieves precision and recall rates of 61.4% and 71.5%, respectively, with an F1-score of 72.5%. The mAP50 and mAP50-95 values reach 75.5% and 66.5%, respectively, surpassing all comparison algorithms across all metrics

## 5. Conclusions

Based on deep learning, this paper investigates pavement crack detection algorithms, proposes optimization improvements to the YOLO11 model, and designs experiments to validate the detection performance of the enhanced model. The research findings are as follows:

1. This paper addresses the challenges in detecting crack targets within complex road scenarios, including weak texture, high aspect-ratio, large scale variations, and strong background interference. Building upon YOLO11 as the baseline, we propose an innovative framework named YOLO11-MBC. The proposed approach incorporates three key improvements: a Feature Fusion Backbone Network (MFFBN) is designed to suppress noise such as oil stains and reflections in the spectral dimension; the BiMCNet neck employs BiFPN combined with MCA cross-modal attention to reconstruct occluded slender cracks in the feature space; and the CGeoCIoU loss with triple penalties for direction, corner points, and contours is introduced to enhance crack boundary perception.
2. Experiments on the public RDD2022 dataset demonstrate that YOLO11-MBC achieves a 22.5% improvement in F1-score and an 8% improvement in mAP50, while retaining 515 FPS on a Tesla A100 with only approximately 9% additional GFLOPs. These results outperform YOLOv8, YOLOv10 and existing crack detectors. Ablation studies and visualizations further confirm that the three proposed modules act synergistically to reduce missed detections and false boxes in complex road scenes.

While the improvements to the pavement crack detection model presented in this study have achieved preliminary results, the following limitations remain:

1. While this study has addressed the multi-scale nature of pavement cracks, it has not yet incorporated multimodal features. Future work will focus on integrating these multimodal characteristics to further enhance the model.
2. Existing datasets mainly consist of images captured under clear weather conditions, with a scarcity of samples from challenging climates such as rain, snow, and fog. Future research will expand the dataset by including images from diverse weather conditions and performing generalization tests to improve the model’s adaptability and robustness in real-world environments.

Future work will continue to refine the model architecture. Since complex and variable weather conditions are commonly encountered in real-world scenarios, subsequent research will focus on enhancing the model’s versatility and robustness. Further improvements will be made to the model structure to boost detection performance, with the ultimate goal of developing a more lightweight, efficient, and practical solution.

## Figures and Tables

**Table 1:** Pavement crack and pothole data.

Type of Dataset	Crocodile Crack	Longitudinal Crack	Transverse Crack	Pothole
Number	19,792	8484	7778	4946

**Table 2:** Hardware configuration table.

Name	Parameters
Operating System	Windows 11
Deep Learning Framework	Pytorch 1.3.11
Programming Language	Python 3.11
CPU	Intel (R) Core (TM) i7-12700H
GPU	RTX3060
RAM	12 GB

**Table 3:** Parameter configuration table.

Parameter	Number
Epoch	150
Learing rate	0.01
Batch Size	32
Learning Rate Decay	0.0005
Momentum	0.937

**Table 4:** Experimental results with different weights of CGeoCIoU.

Methods	*ω*_1_	*ω*_2_	*ω*_3_	AP%	mAP50
Baseline (CIoU)	-	-	-	51.2	69.6
+CGeoCIoU	0.5	0.5	-	53.2	69.6
+CGeoCIoU	**0.6**	**0.4**	**-**	**53.7**	**70.2**
+CGeoCIoU	0.7	0.3	-	53.0	69.6
+CGeoCIoU	0.6	0.4	0.6	53.8	70.2
+CGeoCIoU	**0.6**	**0.4**	**0.7**	**54.5**	**71.1**
+CGeoCIoU	0.6	0.4	0.8	53.5	70.2

Bold values indicate the best results among all methods.

**Table 5:** Ablation experiment data sheet.

Model	Add Modle			F1/%	mAP50	Recall/%	GFLOPs	FPS
	MFFM	BiMCNet	CGeoCIoU					
YOLO11	-	-	-	59.2	69.6	55.8	6.5	554
	√	-	-	63.9	71.7	60.3	6.9	545
	-	√	-	61.9	72.1	60.9	6.7	523
	-	-	√	64.8	71.1	61.8	6.6	549
	√	√	-	71.3	74.6	68.9	7.0	521
	√		√	68.9	73.2	66.4	6.8	537
		√	√	70.2	73.6	66.1	7.0	541
	√	√	√	**72.5**	**75.5**	**71.5**	**7.1**	**515**

FPS measured on Tesla A100 for relative comparison; RTX 3060 yields 73 FPS. Field camera limited to 30 FPS, so no surplus.

**Table 6:** Real comparison data table.

Algorithm	F1/%	mAP50	mAP50-95	Precision/%	Recall/%
YOLOv8	60.6 ± 0.4	59.7 ± 0.4	32.6 ± 0.3	52.7 ± 0.6	50.4 ± 0.5
YOLOv10	58.9 ± 0.3	60.1 ± 0.5	37.4 ± 0.4	55.2 ± 0.5	51.9 ± 0.4
YOLO11	59.2 ± 0.3	69.6 ± 0.4	54.8 ± 0.3	57.4 ± 0.4	55.8 ± 0.6
YOLO11-MBC	**72.5** ** ± 0.2**	**75.5** ** ± 0.3**	**66.5** ** ± 0.3**	**61.4** ** ± 0.5**	**71.5** ** ± 0.4**

Bold values indicate the best results among all methods

## Data Availability

The data are part of an ongoing study, so it is not currently convenient to publish the code and data. However, we welcome other researchers discussing this with us.
